# The use of intramedullary reduction techniques in the treatment of irreducible intertrochanteric femoral fractures with negative medial cortical support

**DOI:** 10.3389/fsurg.2024.1391718

**Published:** 2024-05-13

**Authors:** Xiaowen Huang, Qiang Zuo, Hao Zhou, Tianrun Lv, Jiuxiang Liu

**Affiliations:** Department of Orthopedics, First Affiliated Hospital of Nanjing Medical University (Jiangsu Provincial People’s Hospital), Nanjing, China

**Keywords:** irreducible intertrochanteric fractures, intramedullary reduction, femoral fractures, negative medial support, cortical support techniques

## Abstract

**Objective:**

To explore the clinical efficacy of intramedullary reduction techniques for irreducible intertrochanteric femoral fractures with negative medial cortical support.

**Methods:**

A retrospective analysis was conducted on 69 patients with irreducible intertrochanteric femoral fractures with negative medial cortical support treated in the Department of Orthopedics at Jiangsu Province Hospital (The First Affiliated Hospital of Nanjing Medical University) from July 2019 to December 2021. Patients were divided into Group A and Group B. Group A (experimental group) consisted of 36 cases with an average age of 76.2 ± 5.9 years, while Group B (control group) comprised 33 cases with an average age of 76.6 ± 6.3 years. Group A received treatment using intramedullary reduction techniques, while Group B received treatment using traditional extramedullary reduction techniques. Both groups achieved anatomic reduction of the medial cortex or slight positive support. Surgical duration, intraoperative fracture reduction time, intraoperative bleeding, intraoperative fluoroscopy time, fracture reduction quality, fracture healing, postoperative neck-shaft angle loss, femoral neck shortening, and hip joint functional recovery score (FRS) were compared between the two groups.

**Results:**

All patients were followed up for an average of 13.8 months. Group A showed superior outcomes compared to Group B in surgical duration, intraoperative fracture reduction time, intraoperative bleeding, intraoperative fluoroscopy time, fracture reduction quality, fracture healing, postoperative neck-shaft angle loss, and femoral neck shortening (*P *< 0.05). Hip joint function assessed by functional recovery score was better in Group A than Group B at 1 and 3 months postoperatively (*P *< 0.05), with no significant statistical difference at other time points (*P *> 0.05).

**Conclusion:**

For irreducible intertrochanteric femoral fractures with negative medial cortical support, intramedullary reduction techniques used during surgery demonstrated simplicity, significant reduction in surgical duration, decreased intraoperative bleeding, fewer amounts of intraoperative fluoroscopy, improved fracture reduction quality, and reduced surgical complexity. Further clinical research and application are warranted.

## Introduction

Intertrochanteric femoral fractures are common hip fractures among elderly individuals, with high incidence of complications and mortality when using conservative treatment. Early surgical fixation has become the preferred treatment option (1, 2). However, elderly patients often have multiple systemic comorbidities such as cardiovascular, respiratory, and endocrine conditions. They pose high anesthesia risks and exhibit poor surgical tolerance. Therefore, minimizing surgical trauma, avoiding complex procedures, reducing surgical duration and intraoperative bleeding, and accomplishing fracture reduction and fixation using the simplest and most practical methods play a crucial role in the rapid recovery of elderly patients (1–5). Irreducible intertrochanteric femoral fractures refer to fractures that can not be reduced satisfactorily by conventional traction, abduction, adduction and internal rotation (6, 7). Irreducible intertrochanteric femur fractures with negative medial cortical support occur when the proximal femoral segment gets wedged into the femoral medullary cavity in the coronal plane, forming a torsional effect, rendering the medial cortex in a negative-supporting state. Conventional traction and internal rotation reduction methods are ineffective in unlocking this situation. Forceful unlocking often leads to complete fracture displacement, evolving into a multi-planar irreducible intertrochanteric femur fracture. In the past, this kind of fracture often needed the use of vascular forceps, bone hooks, bone stripping and other tools to pry and pull the fracture end with the assistance of limited incision or small incision, which is time-consuming and laborious. Additionally, maintaining reduction during surgery was challenging, resulting in poor fracture reduction quality (5, 8–13). Therefore, in this study, the displacement characteristics and factors affecting reduction in this type of fracture was analyzed. By employing intramedullary nails as reduction tools and utilizing intramedullary reduction techniques, surgical trauma was effectively reduced, fracture reduction quality was improved, and satisfactory clinical outcomes were achieved. The findings of this study are reported as follows.

## Objects and methods

### Inclusion-exclusion criteria

Inclusion Criteria: (1) Diagnosis of unilateral fresh intertrochanteric femur fracture. (2) Age >65 years. (3) Emergency admission within 24 h post-injury. (4) Intraoperative attempts of conventional traction, abduction, adduction, internal rotation, and other maneuvers fail to achieve reduction. The fracture end presents negative medial cortical support, confirmed as irreducible intraoperative femur fracture with negative medial cortical support under C-arm x-ray fluoroscopy machine. (5) The experimental group is treated with intramedullary nails as reduction tools using intramedullary reduction techniques for fracture reduction. The control group is managed with extramedullary reduction techniques (including but not limited to bone hook traction, vascular forceps and periosteal elevators, temporary fixation with multiple Kirschner wires, and reduction with limited incision). (6) Patients and their families have high compliance and cooperate with treatment and follow-up.

Exclusion Criteria: (1) Patients with severe medical complications, definite surgical contraindications, ASA grade IV or above, could not tolerate surgery. (2) Presence of severe mental illness. (3) Open, old, or pathological fractures. (4) Concurrent ipsilateral hip infection, tumor, rheumatic autoimmune diseases, or history of hip surgery. (5) Inability of patients and family members to comply with follow-up for various reasons.

### Clinical data

A retrospective analysis was conducted on 69 cases of irreducible subtrochanteric femur fractures with negative medial cortical support, admitted to the Orthopedic Trauma Department of Jiangsu Provincial Hospital (First Affiliated Hospital of Nanjing Medical University) from July 2019 to December 2021. All patients were admitted within 24 h post-injury and were enrolled in the Elderly Hip Fracture Fast Track program, with preoperative waiting times of less than 48 h. Based on different methods of fracture reduction during surgery, these 69 patients were divided into Group A and Group B. Group A (experimental) consisted of 36 cases with an average age of 76.2 ± 5.9 years, while Group B (control) included 33 cases with an average age of 76.6 ± 6.3 years. Intraoperatively, C-arm x-ray fluoroscopy confirmed irreducible intertrochanteric femur fractures with negative medial cortical support in all cases. Group A underwent fracture reduction using intramedullary reduction techniques followed by PFNA internal fixation, while Group B underwent fracture reduction using extramedullary reduction techniques followed by PFNA internal fixation. The 69 patients presented various internal medical complications: 55 cases had cardiovascular diseases such as hypertension, coronary artery disease, and a history of myocardial infarction with PCI stent implantation; 18 cases had neurological disorders including lacunar infarction or minor stroke; 25 cases had respiratory system diseases such as chronic obstructive pulmonary disease, emphysema or pulmonary heart disease; 28 cases had type 2 diabetes; 16 cases had gastrointestinal diseases like peptic ulcers or gastritis, and 9 cases had a history of tumors. All patients underwent detailed medical history inquiries, preoperative x-ray, and hip CT examinations, and signed the consent form before operation to obtain the informed consent of patients and their families. There were no statistically significant differences in demographic data (gender, age, injured side, ASA grade, AO/OTA classification, Evans-Jensen classification, cause of injury, time from injury to hospital admission) between Groups A and B (*P* > 0.05, see Table 1). This study was approved by the Ethics Committee of the First Affiliated Hospital of Nanjing Medical University (Ethical Approval Number: 2019-SRFA-458), and all patients provided informed consent prior to their participation.

**Table 1:** Comparison of baseline data between Group A and Group B.

Group	Number of cases	Gender		Age (*x* ± *s*, years)	Injury side		ASA grade			AO/OTA classification		Evans-Jensen classification			Causes of injury		Time from injury to hospital admission (h)
		Male	Female		Left	Right	II	III	IV	31A1	31A2	III	IV	V	Falls	Automobile accident	
Group A	36	17	19	76.2 ± 5.9	19	17	9	17	10	23	13	9	16	11	29	7	7.6 ± 0.9
Group B	33	15	18	76.6 ± 6.3	16	17	8	14	11	19	14	7	17	9	27	6	7.4 ± 1.3
*t*/*χ*^2^ value		0.0216		−0.2723	0.1269		0.2668			0.2881		0.3505			0.0180		0.7482
*P* value		0.883		0.786	0.722		0.875			0.591		0.839			0.893		0.457

### Treatment methods

After emergency admission through the Hip Fracture Fast Track, all patients were conventionally given traction and immobilization of femoral condyles or tibial tuberosity, and anticoagulation with low molecular weight heparin. Prioritized preoperative examinations were arranged, followed by multidisciplinary consultations to regulate the patients’ underlying medical conditions, maintaining preoperative internal stability. Standard preoperative blood preparation and ICU bed placement were also conventionally conducted.

**Figure 1:**
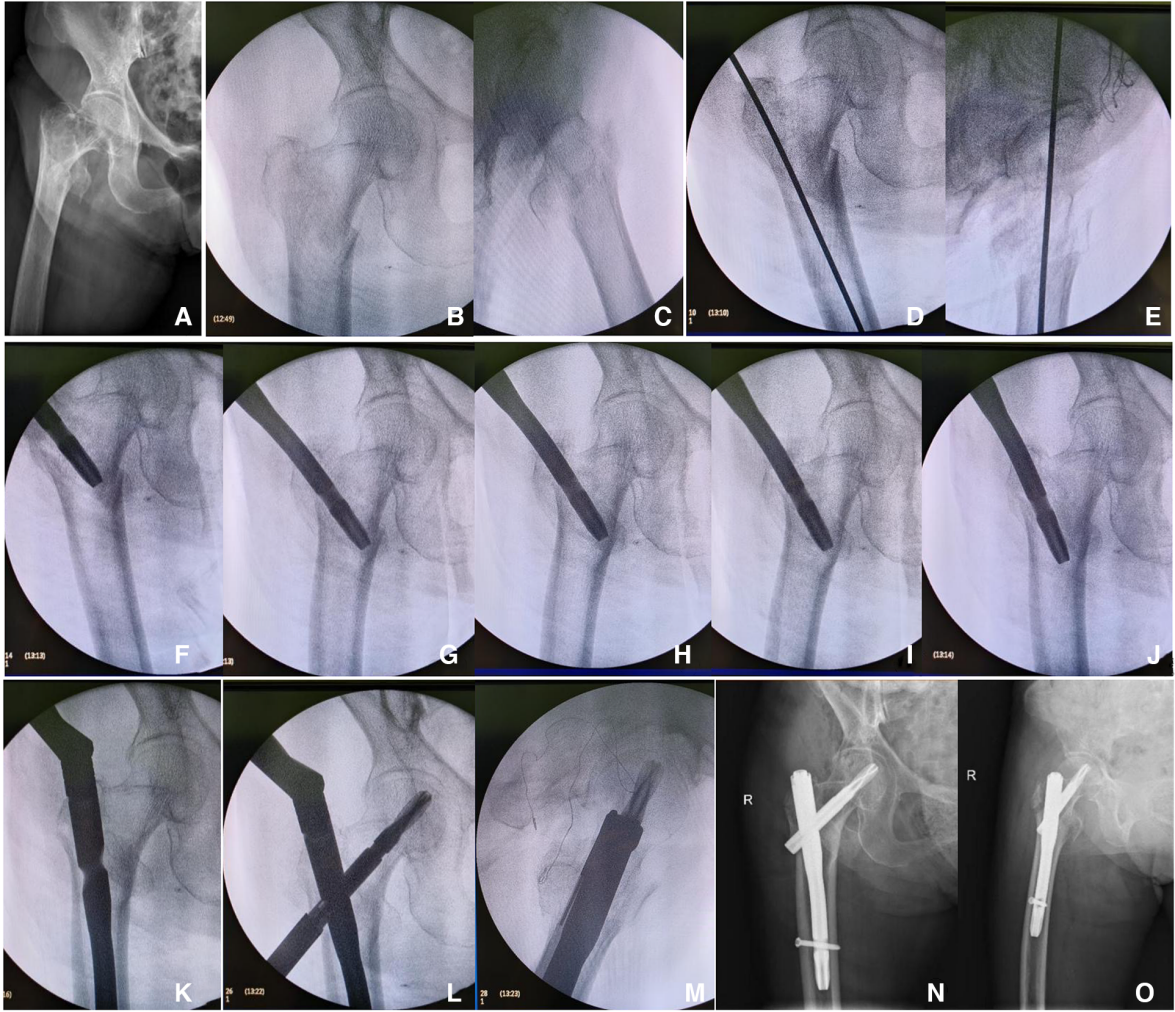
Mrs. Zhang, Female, 82 years old. (**A**) Preoperative x-ray in the anteroposterior position; (**B**,**C**) intraoperative confirmation by fluoroscopy on the C-arm machine of irreducible intertrochanteric femoral fracture with negative medial cortical support; (**D**,**E**) anteroposterior and lateral positioning of the nail entry point, inserting the guide wire, and preparing for proximal reaming; (**F**–**K**) intraoperative utilization of intramedullary reduction for irreducible fracture reduction, converting the negative medial cortex support to medial cortex anatomical reduction; (**L**,**M**) post-reduction fixation with PFNA; (**N**,**O**) postoperative x-ray in anteroposterior and lateral views showing anatomical reduction of the fracture.

General anesthesia was administered to all patients. The surgical position was supine, with the hip of the injured side slightly elevated, and there was no need to place the healthy side in the lithotomy position. Under C-arm x-ray monitoring, routine maneuvers such as traction, abduction, adduction, and internal rotation were initially performed. Despite adequate reduction in the lateral position, complete reduction in the anteroposterior plane was not achieved, indicating coronal plane irreducible fractures. The femoral head-neck fragment was jammed into the medullary cavity of the femoral shaft, with the medial cortex of the head-neck fragment located above the medial cortex of the femoral shaft, demonstrating negative medial cortical support. Therefore, irreducible intertrochanteric femoral fracture with negative medial cortical support was intraoperatively diagnosed. In Group A, after disinfection and draping, a 3–5 cm incision was made at the intersection of the femoral axis and the anterior superior iliac spine. The gluteal muscles were bluntly dissected, and after positioning the trochanterion, a guide wire was inserted under lateral fluoroscopy. After performing a proximal incision to expand the medullary cavity, a PFNA of appropriate size was selected based on the medullary cavity's dimensions. Then, under the guidance and monitoring of a C-arm x-ray machine, the intramedullary nail was slowly inserted into the fracture end, specifically targeting the area of negative support of the medial cortical bone of the femur. Subsequently, the surgeon forcefully used the distal tip of the intramedullary nail to extrude the femoral head-neck fragment jammed into the femoral shaft's medullary cavity, and positioned the fragment on the femoral calcar. The objective of this procedure was to align the medial cortex of the head-neck fragment with the medial cortex of the femoral shaft, either flush or slightly and medially superior, thereby restoring the continuity of the medial Shenton's line. In other words, by employing the bending action of the intramedullary nail's tip within the medullary cavity, the negative support of the medial cortical bone was transformed into anatomical or slightly positive support, completing the reduction procedure for the irreducible fracture. The PFNA nail was then reinserted into the medullary cavity, ensuring no loss of fracture reduction under fluoroscopy. The head-neck nail guide wire was routinely placed, and a spiral blade of appropriate length was driven to 5 mm below the cartilage, and the distal nail was locked. Fluoroscopy again showed that the fracture reduction was acceptable, the internal fixation was satisfactory, and so the wound was washed and sutured (see Figure 1 for typical cases). In Group B, after disinfection and draping, standard extramedullary reduction techniques were applied with limited incisions or minor incisions, including but not limited to methods such as using a bone hook for traction, vascular forceps and bone scraper for leverage, and temporary fixation with multiple Kirschner wires. These methods required assistance from multiple assistants to maintain fracture reduction, placing higher demands on the assistants and making it prone to difficulties in maintaining reduction or the secondary loss of fracture reduction during intramedullary nail insertion. The subsequent PFNA insertion was similar to the routine operations in Group A. Surgeries for Group A and B were performed by orthopedic surgeons with comparable levels of clinical surgical expertise.

Pain relief, infection control, and anticoagulation therapy were routinely given after operation, attention was paid to changing wound dressing, as well as to the changes observed in hemoglobin and albumin. Patients could commence seated rehabilitation exercises on the second day post-operation. Groups A and B patients have scheduled follow-up visits at our hospital at 1, 2, 3, 6, and 12 months postoperatively. Hip joint anteroposterior and lateral x-ray images were taken to assess fracture healing, hip joint function, and associated complications. Based on fracture healing progress during follow-ups, patients were guided to gradually increase weight-bearing using assistive devices until full weight-bearing.

### Observation indexes and clinical efficacy assessments

General Surgical Conditions: preoperative waiting times, surgical duration, fracture reduction time during surgery, amount of intraoperative fluoroscopy, intraoperative bleeding, proportion of postoperative referral to ICU, and occurrence of complications (such as wound infections, pneumonia hypostatic, bed sore, deep vein thrombosis, internal fixation failure, coxa vara, fracture nonunion, etc.) were recorded for both Group A and Group B.

Evaluation of fracture reduction quality, fracture healing, and proximal femoral radiographic parameters: (1) Utilizing Kim's criteria for assessing the quality of intertrochanteric femoral fracture reduction (14): (a) Anatomical reduction or mild valgus reduction on anteroposterior x-rays, angulation within 20 degrees on lateral x-rays. (b) Less than 1 cortical thickness of medial cortical contact on anteroposterior x-rays. (c) Less than 1 cortical thickness of anterior cortical contact on lateral x-rays. Meeting all three conditions constitutes an excellent reduction; meeting condition “a” along with either “b” or “c” constitutes a good (acceptable) reduction, while failing to meet condition “a” implies a poor reduction. (2) Fracture healing will be evaluated based on callus formation observed on x-rays. (3) Proximal femoral radiographic parameters include (a) femoral neck-shaft angle (angle between the axis of the femoral neck fragment and the medullary cavity axis of the femoral-shaft) and (b) femoral neck length (distance from the midpoint of the femoral head along the head-neck axis to the medullary cavity axis). Comparisons will be made between immediate postoperative parameters and final follow-up parameters to calculate changes in femoral neck-shaft angle and femoral neck shortening data.

Hip joint function assessment: The functional recovery of hip joints for both Group A and Group B will be evaluated using the Zuckermann's Fracture Recovery Scale (FRS) for elderly patients with hip fractures (15).

### Statistical analysis

Statistical analysis was performed using the Stata 15.0 software, and numerical variables were represented as mean ± standard deviation ($\bar{x}\pm s$). Normality tests were conducted for all numerical variables, and independent sample *t*-tests were applied for continuous numerical variables that met the criteria of normal distribution between Group A and Group B. For categorical variables, chi-square tests or Fisher's exact probability method were used, with a significance level (*α*) set at 0.05.

## Results

The results indicate that both Group A and Group B patients were followed up for a duration ranging from 12 to 20 months, with an average follow-up period of 13.8 months. There were no cases lost to follow-up. During follow-up, one patient in Group A died due to infection with COVID-19 15 months post-surgery.

Comparing the preoperative waiting time between Groups A and B showed no significant statistical difference (*P *> 0.05). However, Group A exhibited significant advantages over Group B in terms of surgical duration, fracture reduction time during surgery, amount of intraoperative fluoroscopy, intraoperative bleeding, and proportion of postoperative referral to ICU (*P *< 0.05), demonstrating statistical significance. Five cases of iatrogenic lateral wall fractures were observed postoperatively in Group A, and all concentrated near the apex of the greater trochanter compared to Group B (0 case), with statistical significance (*P *< 0.05) (Table 2).

**Table 2:** Comparison of general surgical conditions in Group A and Group B.

Group	Number of cases	Preoperative waiting time (h)	Surgical duration (min)	Fracture reduction time during surgery	Intraoperative fluoroscopy (times)	Intraoperative bleeding (ml)	Postoperative transfer to ICU
Group A	36	26.6 ± 3.9	26.2 ± 7.9	9.5 ± 2.2	23.9 ± 4.1	103.1 ± 17.5	2
Group B	33	27.5 ± 4.3	49.3 ± 6.5	32.1 ± 6.5	41.2 ± 5.8	212.5 ± 23.2	8
*t*/*χ*^2^ value		−0.9117	−13.1934	−19.6792	−14.4007	22.2283	4.8516
*P* value		0.365	<0.001	<0.001	<0.001	<0.001	0.028

Group A demonstrated significant superiority over Group B in fracture reduction quality, fracture healing duration, loss of neck-shaft angle, shortening of the femoral neck, time to achieve full weight-bearing walking, and incidence of complications (*P* < 0.05), indicating statistically meaningful differences (Table 3).

**Table 3:** Comparison of fracture reduction quality, fracture healing, imaging parameters of proximal femur and complications between Groups A and B.

	Number of cases	Kim's fracture reduction quality			Fracture healing (w)	Neck-shaft angle loss (°)	Femoral neck shortening (mm)	Full weight-bearing walking (m)	Complications			
		Excellent	Acceptable	Poor					Wound infection	Internal fixation Failure	Coxa Vara	Fracture nonunion
Group A	36	35	1	0	13.3 ± 0.7	0.9 ± 0.2	1.7 ± 0.4	4.7 ± 0.4	0	0	0	0
Group B	33	26	7	0	14.8 ± 1.5	1.9 ± 0.6	2.9 ± 0.6	5.9 ± 0.7	2	0	1	0
*t*/*χ*^2^ value		4.4817			−5.3957	−9.4491	−9.8503	−8.8352	Fisher			
*P* value		0.034			<0.001	<0.001	<0.001	<0.001	<0.001			

Regarding the hip joint function evaluated using the FRS scores, there were no significant statistical differences between Groups A and B before injury, preoperatively, or at 12 months postoperatively (*P* > 0.05). However, at 1 and 3 months post-surgery, Group A showed superiority over Group B (*P* < 0.05), indicating statistically significant differences (Table 4).

**Table 4:** Comparison of FRS scores of hip joint function between Group A and Group B.

		Group A (*n* = 36)	Group B (*n* = 33)	*T/χ*^2^ value	*P* value
	Before injury	83.8 ± 5.7	83.3 ± 6.2	0.3490	0.728
	Before operation	26.4 ± 4.2	27.1 ± 3.7	−0.7318	0.467
Hip function FRS score	1 month after operation	56.6 ± 3.3	51.5 ± 4.1	5.7137	<0.001
	3 months after operation	61.2 ± 5.6	58.5 ± 3.6	2.3581	0.0213
	12 months after operation	84.5 ± 6.9	84.1 ± 7.3	0.2340	0.816

## Discussion

Currently, surgical treatment has become the preferred option for elderly intertrochanteric femoral fractures. However, elderly patients often have concurrent internal medical conditions, poor tolerance to anesthesia and surgery, and larger surgical traumas, leading to a potential imbalance in their internal environment and resulting in severe postoperative complications. Therefore, on the premise of ensuring the quality of fracture reduction and firm internal fixation, minimally invasive operation should be used to reduce surgical trauma, reduce surgical duration and intraoperative bleeding, so as to significantly improve the prognosis of elderly patients (1, 3, 4).

Irreducible intertrochanteric femoral fractures are those in which satisfactory reduction cannot be achieved through conventional reduction procedures and often require varying degrees of incision reduction. Several previous studies (6, 7) have respectively described their classification and treatment principles. According to these classifications, irreducible intertrochanteric femoral fractures with negative medial cortical support belong to coronal plane irreducible fractures. The difficulty in reduction lies in the proximal femoral block wedging into the femoral medullary cavity, forming a torsion band, resulting in a fracture end exhibiting a displacement characteristic with negative medial cortical support in the coronal plane. Forcible unlocking often leads to the head-neck block becoming a multiplanar irreducible fracture, difficult to reduce both in the coronal and sagittal planes under the effects of the iliopsoas and iliofemoral ligaments. It is suggested that the extramedullary reduction techniques, such as bone stripping and temporary fixation with thick Kirschner wire, should be used during the operation. Extramedullary reduction requires limited incision or auxiliary small incisions to place tools like osteoclasis, vascular forceps, bone hooks, or thyroid hooks to perform elevation and traction. This significantly increases surgical trauma, prolongs surgical duration, and leads to intraoperative bleeding. Furthermore, after the surgery, reduction is often difficult to maintain in extramedullary reduction, requiring assistance through instruments or temporary fixation with Kirschner wires, often resulting in secondary displacement of the fracture end during the insertion of the intramedullary nail, reducing the quality of the reduction, and affecting the prognosis (5, 8–13, 16). Therefore, this study analyzed the displacement characteristics of irreducible intertrochanteric femoral fractures with negative medial cortical support and the shortcomings of extramedullary reduction techniques, attempting to use intramedullary reduction techniques for the reduction of this type of fracture, achieving significant clinical effects.

Intramedullary reduction techniques utilize intramedullary nails as reduction tools to manipulate the fracture end in the medullary cavity. It realigns the inner cortex of the head-neck block to be nearly flush with or slightly internally superior to the inner cortex of the femoral shaft, converting the negative support to anatomical reduction or slight positive support, completing the reduction of this type of fracture. Compared to extramedullary reduction techniques, intramedullary reduction techniques have distinct advantages: (1) No limited incisions or auxiliary small incisions are needed, no requirement for external reduction instrument assistance, and all reduction operations are conducted intramedullary. This avoids the impact of extramedullary reduction operations on the blood supply to the fracture end. (2) The intramedullary reduction is simple to operate, does not need a plurality of assistants to assist and maintain the reduction, and saves manpower. The operation time is short, and the reduction is generally completed by multiple intramedullary nail maneuvers. After reduction, the main nail is inserted directly without damaging the torsion structure of the fracture end. There is no need for instruments like Kirschner wires to maintain the reduction, effectively preventing secondary displacement of the fracture end during the main nail insertion after extramedullary reduction. (3) High reduction quality; C-arm machines can observe the continuity of the femoral medial Shenton's line from multiple angles, accurately controlling the degree of anatomical reduction and slight positive support. (4) The distal end diameter of the intramedullary nail is thin, and the proximal reaming canal is wide,which generally does not interfere with the entry point during intramedullary reduction. It must be emphasized that when using this technique for reduction, if the valgus angle of the intramedullary nail is excessive, there is a risk that the nail may compress the lateral wall of the femur and cause iatrogenic lateral wall fractures. During the procedure, it is imperative to insert the distal end of the intramedullary nail slowly under fluoroscopic guidance with a reduced valgus angle to minimize the pressure on the lateral wall. However, unfortunately, this is inevitable during the initial learning phase. In our case series, iatrogenic lateral wall fractures occurred in five patients during the early learning curve period. Therefore, intramedullary reduction techniques are simple, time-saving, require fewer assistants, have fewer fluoroscopy needs, result in high-quality fracture reduction, significantly reduce surgical duration and intraoperative bleeding, and can be considered the preferred method for the reduction of irreducible intertrochanteric femoral fractures with negative medial cortical support.

Professor Zhang Shimin's theory of positive inner cortical support in intertrochanteric femoral fractures has gradually gained recognition among peers worldwide since 2014 (17–20). However, it needs to be emphasized that the gold standard for fracture reduction is always anatomical reduction. Previous studies have suggested that the 2 mm error in intraoperative C-arm fluoroscopy machine may cause fractures initially considered anatomically reduced to become negative support after surgery. However, this study believes that the error in C-arm fluoroscopy machine can be corrected by observing the continuity of the femoral medial Shenton's line from multiple angles intraoperatively. If the continuity is observed from multiple angles, anatomical reduction can be accepted without forcibly converting to positive support. Additionally, the strength of cortical bone support to cortical bone is definitely superior to that of cortical bone to cancellous bone, which is one of the reasons why we adhere to anatomical reduction. Furthermore, forcibly reducing the fracture end to positive support using intramedullary reduction techniques may damage the torsion relationship between the blocks, posing a risk of being unable to maintain the fracture reduction.

This study also has several limitations. Firstly, all patients still underwent skeletal traction treatment preoperatively. Thus, in our future clinical practice, we will make revisions according to current guidelines. Secondly, it is a retrospective study lacking prospective controlled randomized controlled trials (RCTs), has a relatively small clinical sample size, a short clinical follow-up time, lacks further biomechanical studies.

## Conclusions

In summary, when facing irreducible intertrochanteric femoral fractures with negative medial cortical support, intraoperative intramedullary reduction techniques for fracture reduction are simple, significantly reduce surgical duration, reduce intraoperative bleeding and fluoroscopy needs, improve fracture reduction quality, and reduce surgical difficulty. This approach is worthy of further clinical research and promotion.

## Data Availability

The original contributions presented in the study are included in the article/Supplementary Material, further inquiries can be directed to the corresponding authors.
